# Clozapine-Induced Rhabdomyolysis in a Patient With Paranoid Schizophrenia: A Case Study Highlighting the Importance of Vigilance in Antipsychotic Therapy and Recurrence During Re-Challenge

**DOI:** 10.1192/bjo.2025.10733

**Published:** 2025-06-20

**Authors:** Nitish Jayanth Kumaraswamy, Srishti Agarwal, Manicavasakar Kathirgamar

**Affiliations:** Central and North West London NHS Foundation Trust, London, United Kingdom

## Abstract

**Aims:** A 45-year-old Afro-Caribbean male with a history of paranoid schizophrenia, hypertension, Gilbert syndrome, epilepsy, vitamin D deficiency, mitral regurgitation, and penicillin allergy was admitted in March 2024 for clozapine titration following mental state deterioration. This was his first clozapine re-challenge since suspected clozapine-induced rhabdomyolysis in October 2022, during which CK levels had risen to 7442 IU/L, necessitating discontinuation.

**Methods:** Clinical findings: During titration in March 2024, CK levels rose to 7096 IU/L. The patient, engaging in vigorous exercise, reported mild myalgia but no severe symptoms or NMS.

Diagnostic focus: Clozapine-induced rhabdomyolysis was suspected. IV hydration was initiated, and CK levels decreased to 1500 IU/L after two days but later rose to 4500 IU/L. Clozapine was discontinued, and haloperidol was started, leading to CK normalization.

Second Re-challenge (August 2024): Re-challenged with clozapine, CK levels fluctuated but remained within acceptable limits (1016–833 IU/L), with no symptoms. The patient was encouraged to hydrate and avoid vigorous exercise.

**Results:** Clozapine is effective for treatment-resistant schizophrenia but risks rhabdomyolysis. The mechanism remains unclear but may involve direct myotoxicity or indirect factors like seizures. This case highlights the need for CK monitoring, especially during titration. Elevated CK levels exceeding 5000 IU/L necessitate clozapine discontinuation, IV hydration, and reassessment.

Re-challenging clozapine requires careful evaluation. This patient was successfully re-challenged under close monitoring with tailored CK testing, hydration, and activity recommendations. Vigilance and individualized care minimized risks while maintaining therapeutic benefits.

**Conclusion:** This case underscores the importance of routine CK monitoring, patient education, and interdisciplinary care during clozapine therapy, particularly in those with prior rhabdomyolysis. Personalized treatment strategies and adherence to evidence-based protocols ensure safety and optimize outcomes. Ethnicity- and sex-specific CK reference values should be developed to enhance clinical decision-making. Despite challenges, clozapine re-challenge is feasible with vigilant monitoring and risk mitigation strategies.

